# Approved LXR agonists exert unspecific effects on pancreatic β-cell function

**DOI:** 10.1007/s12020-020-02241-4

**Published:** 2020-03-07

**Authors:** Jonas Maczewsky, Julia Kaiser, Peter Krippeit-Drews, Gisela Drews

**Affiliations:** Institute of Pharmacy, Department of Pharmacology, University of Tübingen, Auf der Morgenstelle 8, 72076 Tübingen, Germany

**Keywords:** Stimulus-secretion coupling, Cytosolic Ca^2+^ concentration, Insulin secretion, LXR, T0901317, GW3965

## Abstract

Novel agonists of the nuclear liver-X-receptor (LXR) are designed to treat metabolic disorders or cancer. The rationale to develop these new drugs is based on promising results with established LXR agonist like T0901317 and GW3965. LXRα and LXRβ are expressed in β-cells, and expression is increased by T0901317. The aim of the present study was to evaluate whether effects of these drugs on β-cell function are specific and reliably linked to LXR activation. T0901317 and GW3965, widely used as specific LXR agonists, show rapid, non-genomic effects on stimulus-secretion coupling of mouse pancreatic β-cells at low µM concentrations. T0901317 lowered the cytosolic Ca^2+^ concentration, reduced or completely inhibited action potentials, and decreased insulin secretion. GW3965 exerted similar effects on insulin secretion. T0901317 affected the production of reactive oxygen species and ATP. The involvement of the classical nuclear LXRs in T0901317- and GW3965-mediated effects in β-cells could be ruled out using LXRα, LXRβ and double knockout mice. Our results strongly suggest that LXR agonists, that are considered to be specific for this receptor, interfere with mitochondrial metabolism and metabolism-independent processes in β-cells. Thus, it is indispensable to test novel LXR agonists accompanying to ongoing clinical trials for acute and chronic effects on cell function in cellular systems and/or animal models lacking classical LXRs.

## Introduction

The liver-X-receptor (LXR) is known to influence cholesterol and lipid metabolism in the liver [1, 2]. The classical subtypes LXRα and LXRβ mediate their effects as nuclear receptors [3]. During the last decade, several effects of LXR on other organs e.g. muscle, adipose tissue and hypothalamus and mitochondria were reported which are important players in the regulation of glucose metabolism (for review see [4]). Besides this, the capability of LXR ligands to influence the concentration of reactive oxygen species (ROS) and to reduce endoplasmic reticulum stress opens new application spectra for LXR agonists particularly against metabolic diseases [5–7]. The suggested pathways are diverse and include cytosolic signal cascades [8], and/or changes in gene expression [9]. In obese animal models of diabetes LXR activation by synthetic agonists improves insulin sensitivity and glucose tolerance [10–12]. The effects were contributed to interference with different pathways in liver, muscle and adipose tissue. In contrast, a study with mice fed a normal diet revealed that T0901317 application in vivo impairs glucose metabolism by decreasing glucose sensitivity and insulin secretion [13]. The findings are not supported by a study with LXRβ-deficient mice: LXRβ^−/−^ mice on low and high fat diet show improved glucose tolerance while LXRβ^−/−^ mice on a standard chow are glucose intolerant [14].

A few studies have tested effects of LXR activation on β-cell function and survival. One study reports that GW3965 protects human islets in vitro against inflammation [15]. The authors suggest LXR activation as a strategy for improving post-transplant islet survival. Another paper shows that T0901317 can protect human, mouse and rat β-cells against palmitate-induced toxicity [16]. Stearoyl-CoA desaturase (SCD) activation seems to be crucial in this cytoprotective effect. Mice lacking LXRα or LXRβ elicit reduced SCD expression. Accordingly, they are more prone to palmitate toxicity. One study with human islets and another one with rat β-cells and MIN6 cells observe increased insulin secretion after LXR activation with T0901317 or GW3965 [17, 18]. The effects are contributed to interaction with enzymes involved in lipid metabolism. In contrast, Meng et al. demonstrate that T0901317 leads to β-cells dysfunction. They show with mouse β-cells and MIN6 cells reduced insulin secretion in response to LXR activation accompanied by decreased oxygen consumption, ATP production and current through L-type Ca^2+^ channels [13].

All these in vivo and in vitro studies were long-term studies with the LXR agonists T0901317 or GW3965. Possible acute or **non**-LXR-mediated effects were not considered in these studies.

Recently, we showed activation of a non-genomic rapid pathway by 10 µM T0901317 that negatively influenced insulin secretion in murine and human pancreatic β-cells [19]. The LXR agonist interfered with stimulus-secretion coupling (SSC) by inhibiting the ATP synthesis probably due to effects on the ROS concentration [19]. In the present study, we challenge the assumption that effects of T0901317 or GW3965 can reliably be attributed to LXR activation. Our results question the specificity of the so-called LXR agonists that provide the basis for the development of novel drugs.

## Research design and methods

### Cell and islet preparation

Details are described in [20]. In brief, mouse islets were isolated by injecting collagenase (0.5–1 mg/ml) into the pancreas, and by handpicking after digestion at 37 °C. Male and female wild type C57Bl/6 mice were used in equal shares. Development of mice with general ablation of LXRα and LXRβ are described in [21]. All LXR knockout (LXR^−/−^) mice are on a C57Bl/6 background and were housed under same conditions. Mice were bred in the animal facility of the Department of Pharmacology at the University of Tübingen. Principles of laboratory animal care (NIH publication no. 85–23, revised 1985) and German laws were followed. Mouse islets were dispersed to single cells and cell clusters, respectively, by trypsin treatment.

### Solutions and chemicals

Recordings of the cytosolic Ca^2+^ concentration ([Ca^2+^]_c_) were performed with a bath solution which contained (mM): 140 NaCl, 5 KCl, 1.2 MgCl_2_, 2.5 CaCl_2_, glucose as indicated, 10 HEPES and pH 7.4 adjusted with NaOH. Same bath solution was used for determination of the mitochondrial membrane potential (ΔΨ), ROS and membrane potential (V_m_) measurements in the perforated-patch configuration. The bath solution was modified by adding 0.1% bovine serum albumin (BSA) and used as incubation medium for ATP measurements. Krebs–Ringer–HEPES (KRH) solution for measurements of insulin secretion was composed of (mM): 120 NaCl, 4.7 KCl, 1.1 MgCl_2_, 2.5 CaCl_2_, glucose as indicated, 10 HEPES, 0.5% BSA and pH 7.4 adjusted with NaOH. Pipette solution for measurements of V_m_ consisted of (mM): 10 KCl, 10 NaCl, 70 K_2_SO_4_, 4 MgCl_2_, 2 CaCl_2_, 10 EGTA, 20 HEPES, 0.27 amphotericin B and pH adjusted to 7.15 with KOH. Islet cell clusters and pancreatic islets were cultured in RPMI 1640 (11.1 mM glucose) enriched with 10% foetal calf serum (FCS) and 1% penicillin/streptomycin.

T0901317 and GW3965 were obtained from Biomol (Hamburg, Germany). Fura-2-AM was purchased from Biotrend (Köln, Germany) and 25-OH cholesterol from Santa Cruz (Heidelberg, Germany). Rhodamine 123 (Rh123), RPMI 1640 medium, FCS, penicillin/streptomycin, 2′,7′-dichlorodihydrofluorescein-diacetate (DCDHF-DA), dihydroethidium (DHE), ATP determination kit and trypsin were from Invitrogen (Karlsruhe, Germany). All other chemicals were purchased from Sigma (Taufkirchen, Germany) or Merck (Darmstadt, Germany) in the purest form available.

### Measurement of [Ca^2+^]_c_

Details are described in [20]. In brief, cells were loaded with 5 µM Fura-2-AM for 35 min at 37 °C. The fluorescence was excited at 340 nm and 380 nm and the emission was measured. [Ca^2+^]_c_ was calculated according to an in vitro calibration. The maximum amplitude of Ca^2+^ oscillations (max. [Ca^2+^]_c_) was taken to compare [Ca^2+^]_c_ under different experimental conditions.

### ATP measurement

Details are described in [19]. 20 islets were kept for 30 min at 37 °C in incubation medium under conditions as indicated. After cell lyses by adding a solution containing 200 mM NaOH and 0.5 mM EDTA, ATP concentration was elicited by the ATP determination kit according to manufactures instructions. Bioluminescence was determined with Luminometer 1253 (Bioorbit, Turku, Finland) in triplicate. Data were normalised to control condition under 15 mM glucose.

### Patch-clamp measurements

V_m_ measurements were recorded with an EPC-9 patch-clamp amplifier using Patchmaster software (HEKA, Lambrecht, Germany) with the perforated-patch configuration in the current clamp mode at a holding current of 0 mA. For determination of V_m_ average plateau potential was evaluated 1 min before solution change. The same time interval was used for determination of action potential frequency.

### Insulin secretion

Details for steady-state incubations are described in [20]. Briefly, batches of five islets in triplicate were incubated in 1 ml KRH for 1 h at 37 °C under conditions as indicated. In experiments in which acute effects were investigated, the agonists were only present during the 1 h secretion measurement. To determine chronic effects, the agonists were absent during the 1 h secretion phase but the islets were pre-incubated for 72 h with agonists as indicated. Insulin was determined by radioimmunoassay (Merck Millipore, Darmstadt, Germany) with rat insulin as standard.

### Measurements of ΔΨ

ΔΨ was measured as Rh123 fluorescence (arbitrary units (a.u.) at 480 nm excitation wave length as described elsewhere [22]. To evaluate the effects, the values were averaged over 150 s at the end of each interval, i.e. before solution change.

### Measurement of ROS

ROS production was measured using the fluorescent dyes DCDHF-DA and DHE. In the cells DCDHF-DA is oxidised to the fluorescent 2′,7′-dichlorofluorescein (DCF), which detects H_2_O_2_ and other ROS species. DHE is oxidised to 2-hydroxyethidium (2-OH-E^+^), which mainly indicates O_2_^−^ formation. After 1 h incubation under conditions as indicated, the islet cell clusters were loaded for 15 min with 20 µM DCDHF-DA and 10 µM DHE, respectively. Fluorescence was excited at 480 nm and the intensity of the emitted light (arbitrary units (a.u.) was measured.

### Statistics

Each series of experiments was performed with islets or islet cell clusters from at least three different mice. Means ± SEM are given for the indicated number of experiments (islet cell clusters or islets). Statistical significance of differences was assessed by a paired Student’s *t* test. Multiple comparisons were made by repeated ANOVA followed by the Student–Newman–Keuls test. *P* values ≤ 0.05 were considered significant.

## Results

### LXR agonists acutely affect SSC in β-cells

**Fig. 1:**
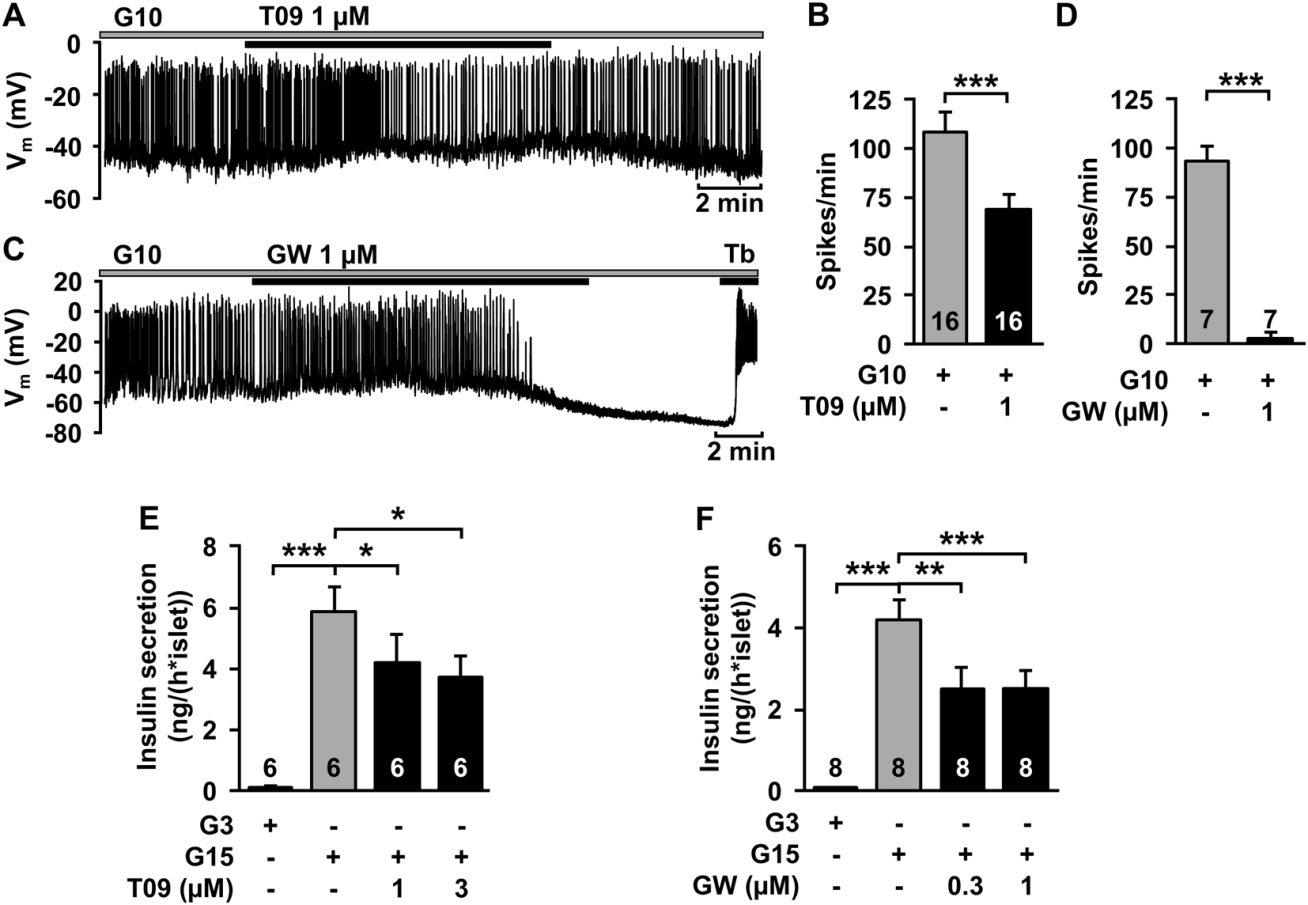
LXR agonists acutely inhibit stimulus-secretion coupling in wild type β-cells. **a** Representative recording of V_m_ with administration of T0901317 (T09). **b** Summary of all experiments. *n* = 16. **c** Representative recording of V_m_ with administration of GW3965 (GW). Tolbutamide (Tb) was given at the end of the experiment to test whether closure of K_ATP_ channels is still possible. **d** Summary of all experiments. *n* = 7. **e** Glucose-induced insulin secretion is reduced by T0901317 compared with control islets in the presence of 15 mM glucose (G15). *n* = 6. **f** GW3965 also decreased glucose-induced insulin secretion. *n* = 8. Experiments were performed with different islet cell clusters or islets from 3–7 mice. *P ≤ 0.05, **P ≤ 0.01, ***P ≤ 0.001

Recently, we made the unexpected observation that 10 µM T0901317 exerts acute effects on SSC in β-cells [19]. Thus, in a first approach we investigated effects of lower concentrations of T0901317 on β-cell function, which are most likely more specific for the LXR, and compared it to another drug which is assumed to be a specific LXR ligand, GW3965. One micromolar T0901317 reduced the frequency of action potentials (Fig. 1a, b). GW3965 (1 µM) mimicked this effect on V_m_ (Fig. 1c, d). As expected from these observations, acute administration of T0901317 (1 µM and 3 µM) and GW3965 (0.3 µM and 1 µM), respectively, inhibited glucose-stimulated insulin secretion (Fig. 1e, f). Metabolic integrity of the β-cells is shown by increased insulin secretion at stimulating glucose concentration (15 mM) compared with a sub-stimulatory concentration (3 mM).

### Chronic administration of LXR agonists inhibits insulin secretion

**Fig. 2:**
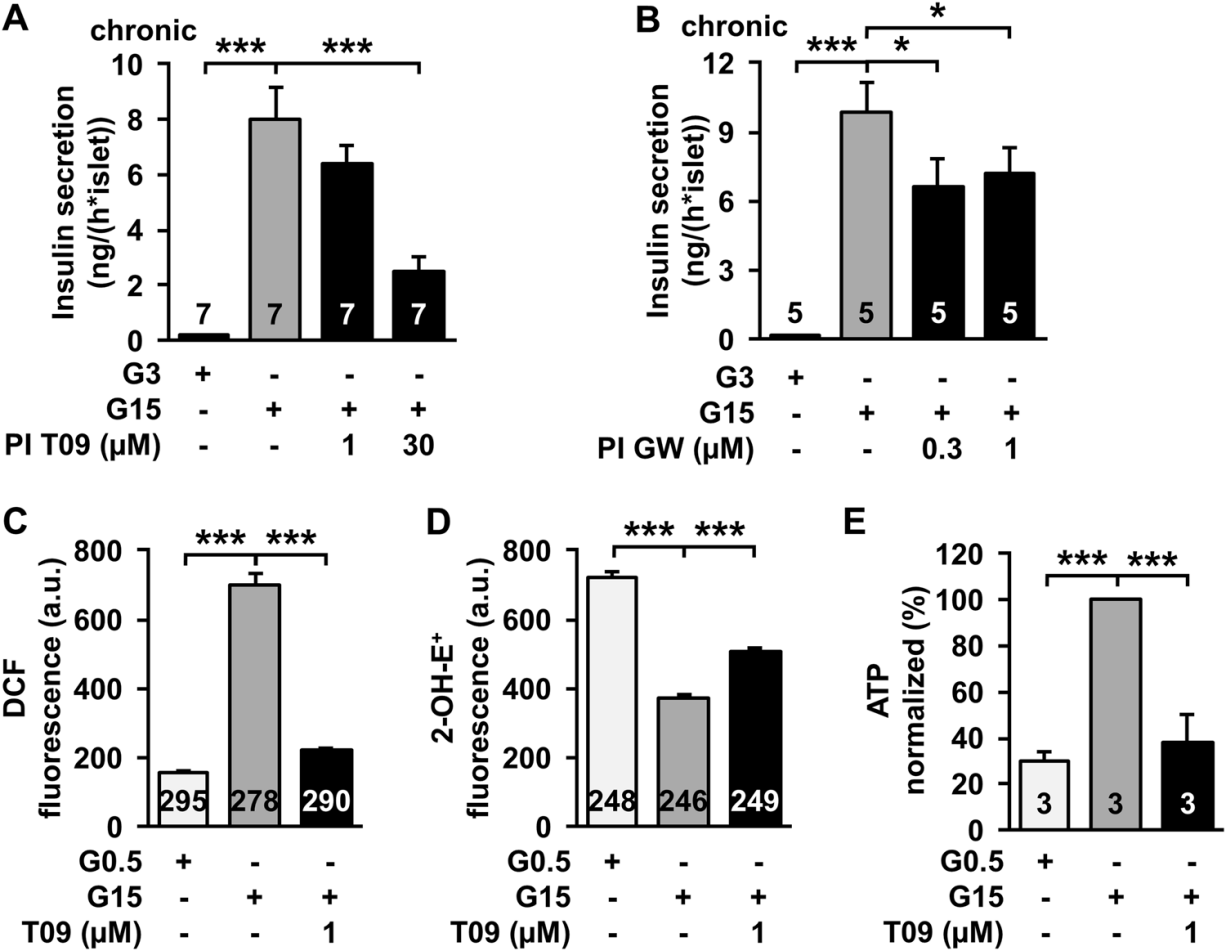
LXR agonists influence β-cell metabolism. **a** Glucose-induced insulin secretion after 72 h pre-incubation (PI) with T0901317 compared with control conditions in the presence of 15 mM glucose. T0901317 was not present during the 1 h of steady-state incubation for the determination of insulin secretion. *n* = 7. **b** Glucose-induced insulin secretion after 72 h pre-incubation with GW3965. GW3965 was not present during the 1 h secretion measurement. *n* = 5. **c** Measurement of DCF-detectable oxidants in islet cell clusters. Increasing the glucose concentration from 0.5 to 15 mM leads to enhanced DCF-fluorescence. Administration of T0901317 in the presence of 15 mM glucose resulted in a massive reduction of DCF-detectable ROS. *n* = 278–295. **d** Determination of 2-OH-E^+^-detectable oxidants in islet cell clusters. Increasing the glucose concentration from 0.5 to 15 mM reduces the fluorescence. T0901317 leads to an increase of 2-OH-E^+^-detectable ROS at high glucose concentration. *n* = 246–249. **e** Measurement of ATP concentration in intact islets. ATP content of islets is significantly reduced by T0901317 in comparison to control islets kept in 15 mM glucose. *n* = 3. Experiments were performed with different islet cell clusters or islets from 3–7 mice. *P ≤ 0.05, ***P ≤ 0.001

In the literature effects of LXR agonists are described solely after chronic administration. Therefore, we performed insulin secretion experiments after 72 h pre-incubation with the same agonists that showed acute effects. Although, no agonists were present during the 1 h secretion measurement, glucose-stimulated insulin secretion was decreased after pre-incubation with T0901317 and GW3965 (Fig. 2a, b). Remarkably, acute and chronic administration of the drugs revealed comparable effects.

### T0901317 influences ATP synthesis and ROS production

The SSC in β-cells is triggered by ATP synthesis due to redox processes within mitochondria. Increasing the glucose concentration from 0.5 to 15 mM led to enhanced DCF fluorescence (mainly H_2_O_2_ production) in islet cell clusters. Administration of T0901317 in the presence of 15 mM glucose resulted in a massive reduction of DCF-detectable ROS. (Fig. 2c). In contrast, enhancing the glucose concentration from 0.5 to 15 mM reduced 2-OH-E^+^-detectable ROS (mainly O_2_^−^) while T0901317 provoked an increase of 2-OH-E^+^-detectable oxidants at high glucose concentration. Likewise, ATP concentration under high glucose condition measured in intact islets was critically reduced by T0901317 (Fig. 2e). Metabolic integrity was shown by increased ATP concentration at stimulating glucose concentration (15 mM) compared with a sub-stimulatory concentration (3 mM).

### LXR agonists interfere with metabolism-independent processes

**Fig. 3:**
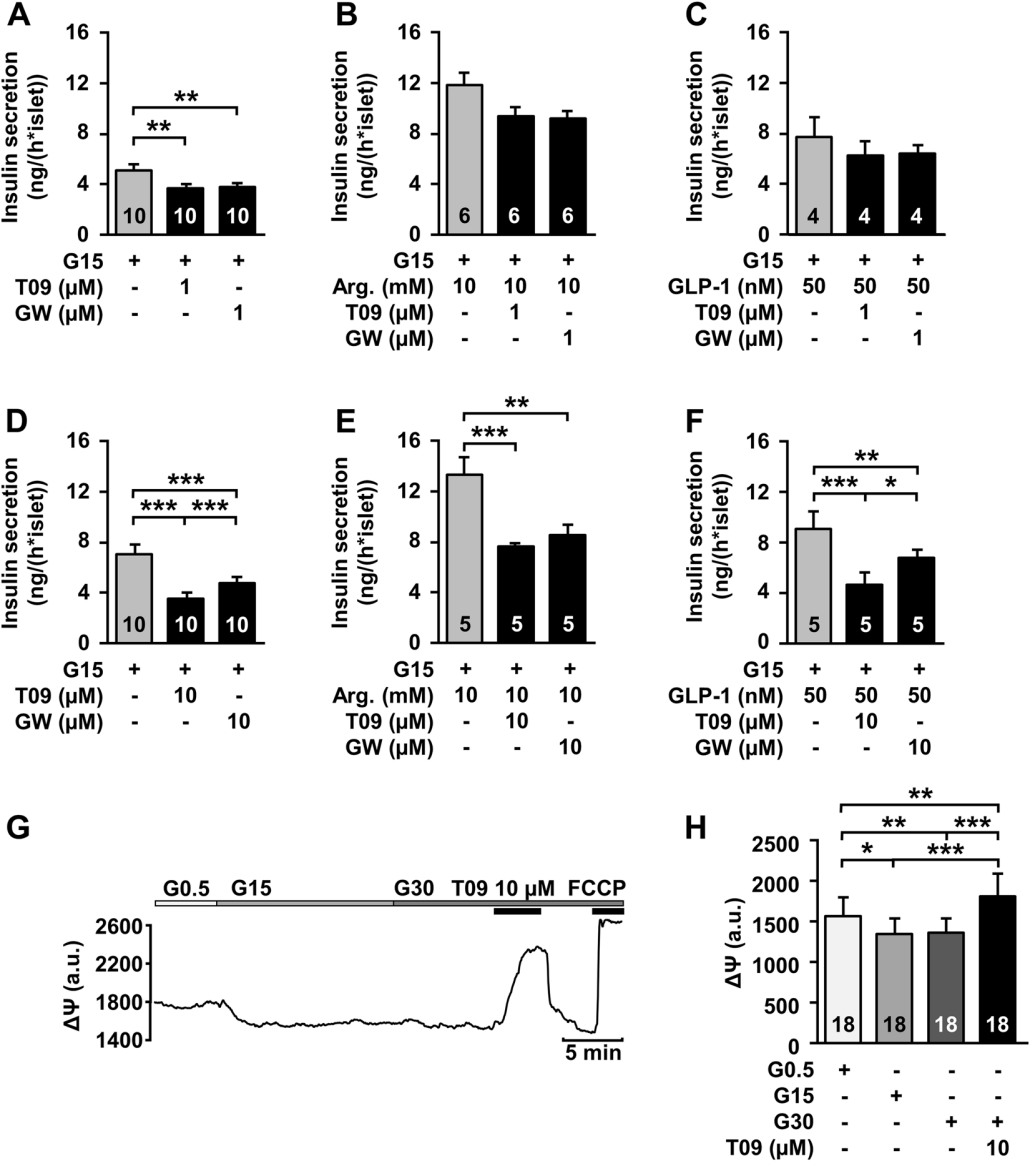
LXR agonists exerts metabolism-independent effects. Influence of 1 µM T0901317 (T09) and 1 µM GW3965 (GW), respectively, on insulin secretion in the presence of 15 mM glucose (**a**, control), 10 mM arginine (**b**) and 50 nM GLP-1 (**c**). *n* = 4–10. The agonists had no significant effects in the presence of arginine or GLP-1. Inhibiting effect of 10 µM T0901317 (T09) and 10 µM GW3965 (GW), respectively, on insulin secretion in the presence of 15 mM glucose (**d**, control), 10 mM arginine (**e**) and 50 nM GLP-1 (**f**). *n* = 5–10. Administration of 10 µM T0901317 on ΔΨ in the presence of 30 mM glucose still reveals a strong depolarisation. **g** Representative recording of ΔΨ with administration of T0901317. Metabolic integrity is shown by a rapid depolarisation by 0.5 µM FCCP. **h** Summary of all experiments. *n* = 18. Experiments were performed with different islet cell clusters or islets from 3–10 mice. *P ≤ 0.05, **P ≤ 0.01, ***P ≤ 0.001

The effects of the LXR agonists on ATP and ROS production point to interference with mitochondrial metabolism. We evaluated whether the agonists could also influence mitochondria-independent β-cell stimulation by arginine or GLP-1. Figure 3a–c shows that arginine as well as GLP-1 strongly attenuated the inhibiting effect of 1 µM T0901317 and 1 µM GW3965, respectively. However, in the presence of higher concentrations of the agonists (10 µM) arginine or GLP-1 could not diminish the inhibitory effect of the drugs (Fig. 3d–f). Interestingly, even 30 mM glucose could not hinder the strong depolarisation of ΔΨ induced by 10 µM T0901317 (Fig. 3g, h), which reflects inhibition of ATP production.

### LXR agonists acutely affect SSC in β-cells of LXR^−/−^ mice

**Fig. 4:**
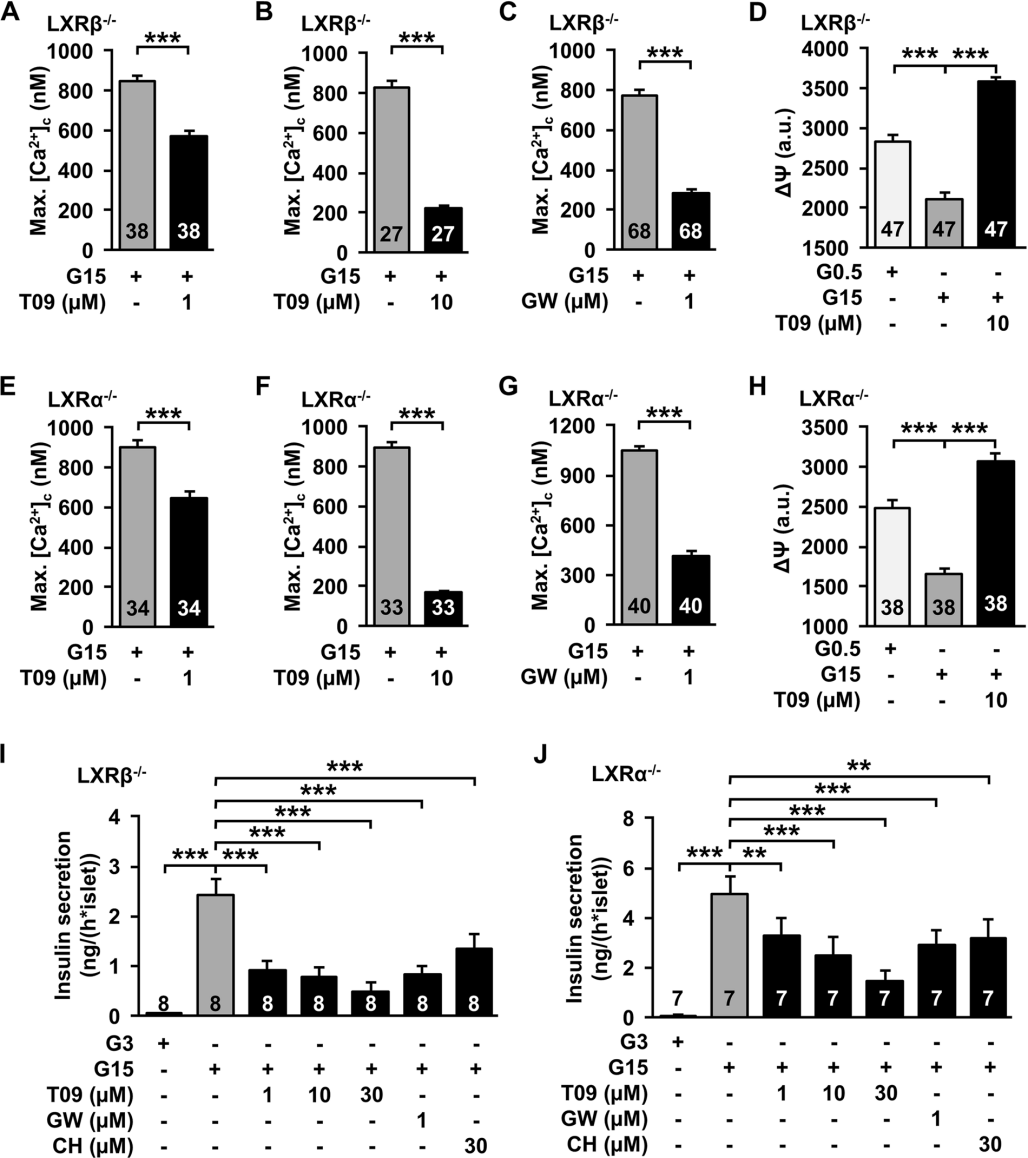
Effects of LXR agonists are present in LXR^−/−^ β-cells in the presence of 15 mM glucose. **a**, **b** T0901317 (1 µM and 10 µM) reduce maximal [Ca^2+^]_c_ in β-cells of LXRβ^−/−^ mice. *n* = 38 and 27. **c** 1 µM GW3965 reduces maximal [Ca^2+^]_c_ in β-cells of LXRβ^−/−^ mice. *n* = 68. **d** 10 µM T0901317 depolarises ΔΨ in islet-cell clusters of LXRβ^−/−^ mice. Metabolic integrity is shown by hyperpolarisation of ΔΨ after changing the glucose concentration from 0.5 to 15 mM. *n* = 47. **e**, **f** 1 µM and 10 µM T0901317 reduce maximal [Ca^2+^]_c_ in β-cells of LXRα^−/−^ mice. *n* = 34 and 33. **g** 1 µM GW3965 reduces maximal [Ca^2+^]_c_ in β-cells of LXRα^−/−^ mice. *n* = 40. **h** 10 µM T0901317 depolarises ΔΨ in islet cell clusters of LXRα^−/−^ mice. *n* = 38. **i**, **j** Glucose-induced insulin secretion is reduced by T0901317, GW3965 and 25-OH-cholesterol (CH) in islets from LXRβ^−/−^ and LXRα^−/−^ mice. *n* = 8 and 7. Experiments were performed with different islet cell clusters or islets from 3–8 mice. **P ≤ 0.01, ***P ≤ 0.001

Similar effects of different LXR agonists suggest that the LXR is indeed targeted by the drugs. We tested the effects of T0901317 and GW3965 in β-cells isolated from LXRβ^−/−^ mice. Both agonists decreased maximal [Ca^2+^]_c_ (max.[Ca^2+^]_c_) and T0901317 depolarised ΔΨ under stimulating glucose condition (Fig. 4a–d). As the results did not show any impact of the LXRβ^−/−^, the measurements were repeated with β-cells isolated from LXRα^−/−^ mice. In LXRα^−/−^ β-cells T0901317 and GW3965 decreased max.[Ca^2+^]_c_ (Fig. 4e–g). Like the previous observation, T0901317 depolarised ΔΨ in these knockout islet cell clusters (Fig. 4h). Accordingly, acute administration of T0901317 and GW3965 inhibited glucose-stimulated insulin secretion in pancreatic islets of LXRα^−/−^ and LXRβ^−/−^ mice, respectively (Fig. 4i, j). In these series of experiments we tested in addition the endogenous LXR agonist 25-OH-cholesterol, which revealed similar results (Fig. 4i, j).

**Fig. 5:**
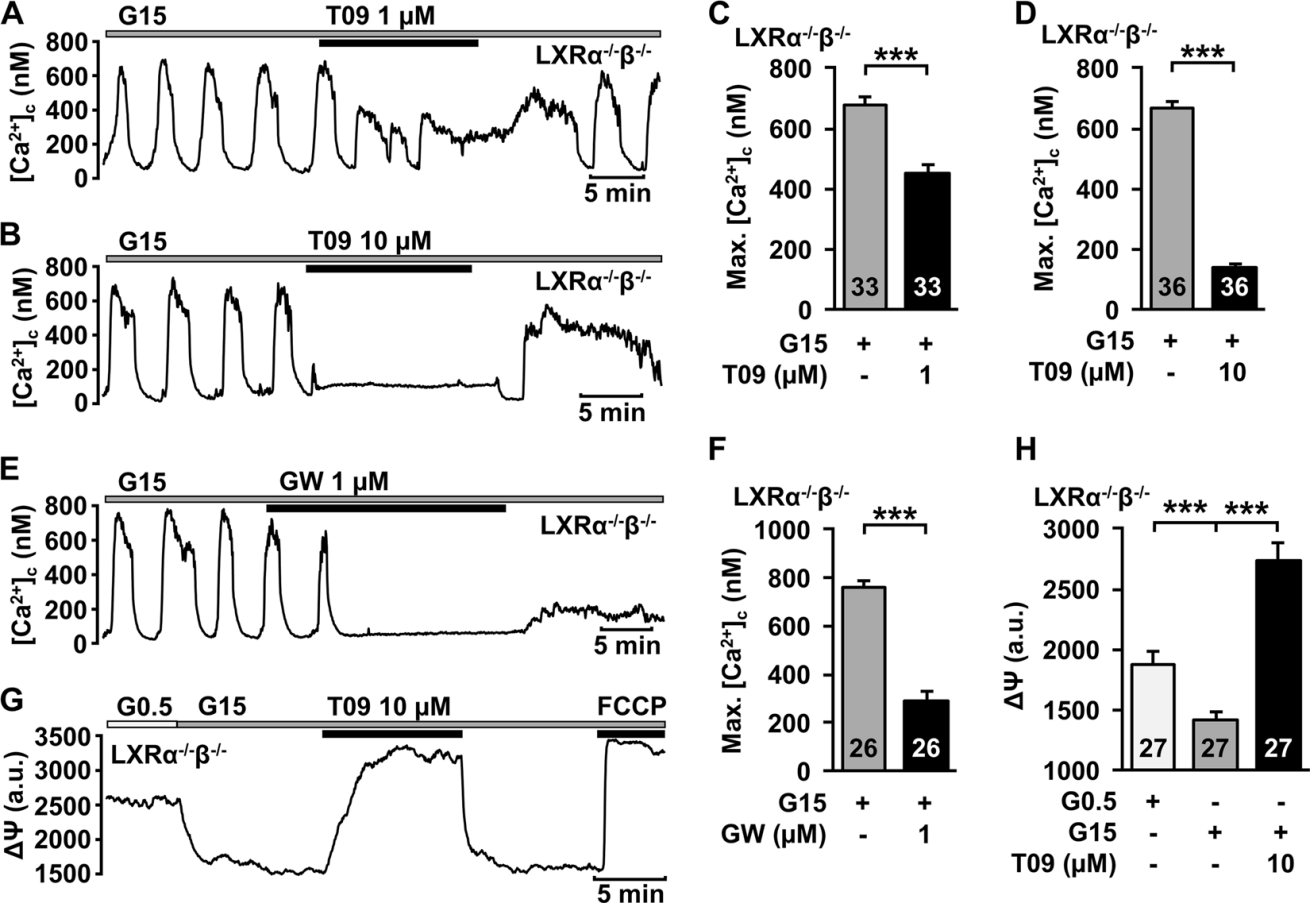
Acute effects of LXR agonists are present in LXRα^−/−^β^−/−^ β-cells. **a**, **b** Representative measurements showing inhibition of glucose-induced oscillations of [Ca^2+^]_c_ by 1 µM T0901317 (**a**) and 10 µM T0901317 (**b**) in the presence of 15 mM glucose in β-cells of LXRα^−/−^β^−/−^ mice. **c**, **d** 1 µM and 10 µM T0901317 reduces maximal [Ca^2+^]_c_ in β-cells of LXRα^−/−^β^−/−^ mice. *n* = 33 and 36. **e** Representative measurement showing inhibition of glucose-induced oscillations of [Ca^2+^]_c_ by 1 µM GW3965 in the presence of 15 mM glucose in β-cells of LXRα^−/−^β^−/−^ mice. **f** 1 µM GW3965 reduces the maximal [Ca^2+^]_c_ in β-cells of LXRα^−/−^β^−/−^ mice. *n* = 26. **g** Representative measurement of ΔΨ in islet cells of LXRα^−/−^β^−/−^ mice. Increasing the glucose concentration from 0.5 to 15 mM leads to hyperpolarization of ΔΨ due to enhanced ATP production. Administration of T0901317 results in rapid and reversible depolarisation of ΔΨ. Metabolic integrity is shown by a rapid depolarisation by 0.5 µM FCCP. **h** Summary of all experiments of this series. *n* = 27. Experiments were performed with different islet cell clusters from three mice. ***P ≤ 0.001

To exclude a possible compensation of LXRα or LXRβ by higher protein expression of the corresponding receptor in the respective knockout model, a double knockout mouse model with the concurrent deletion of LXRα and LXRβ was used. In β-cells of these mice the effect of T0901317 and GW3965 on max.[Ca^2+^]_c_ was still present (Fig. 5a–f). The higher concentration of T0901317 completely abolished glucose-dependent Ca^2+^ oscillations (Fig. 5b). The drop in [Ca^2+^]_c_ directly after removal of T0901317 points to ATP-dependent SERCA activation after ATP depletion [19]. Accordingly, T0901317 depolarised ΔΨ in LXRα^−/−^β^−/−^ islet cell clusters under high glucose condition (Fig. 5g, h).

## Discussion

### Reputed LXR agonists acutely affect SSC by non-genomic actions

Several studies show effects on metabolism after long exposure to reputed LXR agonists including T0901317 and suggest genomic actions [9–13, 15–18]. In the present study 1 µM T0901317 and GW3965 acutely inhibited SSC in pancreatic β-cells within a few minutes. Evidently, the observed effects were not limited to high concentration of the agonists (T0901317 in [19]), but also occur with lower concentrations of the drugs (T0901317 and GW3965 in this study) that are assumed to be more specific. A genomic pathway is highly unlikely due to the fast onset of the effects seen in patch-clamp experiments and Ca^2+^ measurements.

Remarkably, T0901317 affects platelet aggregation of nucleus free thrombocytes [23–25]. Furthermore, rapid effects of T0901317 were observed in colon cancer cells [26, 27]. These observations support our findings that LXR agonists can act in a non-genomic manner, independent from transcription processes in the nucleus.

Meanwhile, non-genomic actions of ligands formerly classified as typical agonists of nuclear receptors are well accepted; e.g. oestrogens activate cytosolic signal cascades in β-cells [28–30]. Interestingly, the nuclear oestrogen receptor (ER) can translocate to the cell membrane, where it triggers rapid signalling [31, 32]. Moreover, 17β-oestradiol can directly modify K_ATP_ channel activity and interfere with the SUR1 channel subunit in β-cells [29, 33]. Furthermore, a rapid, cytosolic pathway that alters K_ATP_ activity in β-cells is shown for the nuclear farnesoid-X-receptor [34].

### The classical nuclear LXR does not activate the acute pathway induced by T0901317 and GW3965 in β- cells

The role of LXRs in β-cell function is not completely understood. Data obtained with WT mouse or human islets in which synthetic LXR agonists have been used and LXR-deficient islets as well as in vivo and in vitro findings reveal many inconsistencies (see Introduction). There is not even unity whether LXR activation leads to stimulatory or inhibitory effects in vitro and to beneficial or detrimental effects in vivo. One reason for this complex situation may be that LXRs can exert opposite function in lean and obese animals, i.e. the function depends on the dietary state [14]. A similar function dependency on the nutritional situation has been described for the FXR earlier [35]. Another reason for contradictory findings may be the fact that synthetic LXR activators exert rapid, non-genomic, unspecific effects (see below).

We used LXR^−/−^ mice to evaluate the role of classical LXRs in the actions of the reputed LXR agonists. The ubiquitously identified LXRβ does not influence the acute actions of LXR agonists in β-cells, shown by experiments with cells from LXRβ^−/−^ mice. Compared with the LXRβ, the LXRα seems to be less abundant in mouse islets and not detectable in β-cells [36]. We show that the α-subtype also does not participate in the acute effects of GW3965 and T0901317 in β-cells. A third reputed LXR agonist, 25-OH-cholesterol, which is believed to be the endogenous activator of the receptor also suppresses glucose-induced insulin secretion in both genotypes.

To circumvent a possible counter-regulatory upregulation of LXRα in LXRβ^−/−^ cells and vice versa, cells from LXRα^−/−^β^−/−^ mice were used. Even in these cells, GW3965 and T0901317 exerted their inhibitory effects on SSC. The results clearly point to a mechanism independent of the classical LXRs.

The fact that three chemically different so-called LXR agonists act in an analogue manner suggests the presence of a different target with a binding profile like the classical LXR. The rapid effects, most likely evoked by cytosolic signal cascades, point to an action of this non-classical LXR at the membrane or in the cytosol. The paper of Meng et al. [13] and our studies (this and [19]) point to interaction with mitochondria i.e. changes in ROS production and ATP depletion as one possible mode of action in β-cells. The action of low concentrations of T0901317 and GW3965 could be counteracted by increasing insulin secretion with either arginine or GLP-1. This seems to be irrespective of the mechanism because arginine depolarises via influx of positive ions, and GLP-1 by stimulating the amplifying pathway via an increase of the cAMP concentration. The data suggest that low concentrations of the drugs can affect SSC beyond interference with mitochondrial metabolism. However, at higher concentrations (10 µM) of the agonists, arginine and GLP-1 did not prevent the inhibitory effect any more suggesting that at this concentration the effect on mitochondrial metabolism prevails. This assumption is further supported by the fact that increasing the glucose concentration to the maximal depolarising effect of glucose (30 mM) did not suppress the strong depolarisation of ΔΨ provoked by T0901317.

The functionality of mitochondrial processes is essential in all organs and cell systems. Therefore, several effects attributed to LXR in different cell types may be caused by the interaction of LXR agonists with mitochondrial function. As a result, our data question conclusion about the involvement of LXR in physiological processes drawn from studies with T0901317 or GW3965 without use of LXR-deficient cell systems or mice.

### A word of caution

Previous results of studies with established LXR ligands led to the development of new LXR agonists for therapeutic use in cancer and metabolic diseases. Very recently, it has been shown that LXR agonism by GW3965, and the novel LXR ligand RGX-104 enhances the activation of cytotoxic T-lymphocytes in mice and humans with cancer [37]. A clinical phase 1 study was accomplished with the LXR agonist BMS-852927 [38]. The authors draw the conclusion that BMS-852927 exerts beneficial effects on hypercholesterolaemia in humans by activation of the LXR. But they also observed adverse effects e.g. on lipogenesis and neutrophilic granulocytes in healthy volunteers questioning the therapeutic impact [38]. Another first-in-human study with the LXR ligand LXR-623 was performed with healthy participants to test a possible application of LXR agonists in the therapy of arteriosclerosis [39]. In this study, LXR-623 led to increased appearance of adverse events related to the central nervous system [39].

We show unspecific actions of well-established LXR agonists suggesting re-interpretation of previous results. The exact target mediating the observed rapid effects is still unknown. This leads to a more complex assessment of possible risk factors, and side effects of all LXR agonists. Thus, it is indispensable to intensify the research with potential new drugs with respect to acute, LXR-independent effects.
